# DYVIPAC: an integrated analysis and visualisation framework to probe multi-dimensional biological networks

**DOI:** 10.1038/srep12569

**Published:** 2015-07-29

**Authors:** Lan K. Nguyen, Andrea Degasperi, Philip Cotter, Boris N. Kholodenko

**Affiliations:** 1 Systems Biology Ireland, University College Dublin, Belfield, Dublin 4, Ireland; 2 Conway Institute, University College Dublin, Belfield, Dublin 4, Ireland; 3 School of Medicine and Medical Science, University College Dublin, Belfield, Dublin 4, Ireland

## Abstract

Biochemical networks are dynamic and multi-dimensional systems, consisting of tens or hundreds of molecular components. Diseases such as cancer commonly arise due to changes in the dynamics of signalling and gene regulatory networks caused by genetic alternations. Elucidating the network dynamics in health and disease is crucial to better understand the disease mechanisms and derive effective therapeutic strategies. However, current approaches to analyse and visualise systems dynamics can often provide only low-dimensional projections of the network dynamics, which often does not present the multi-dimensional picture of the system behaviour. More efficient and reliable methods for multi-dimensional systems analysis and visualisation are thus required. To address this issue, we here present an integrated analysis and visualisation framework for high-dimensional network behaviour which exploits the advantages provided by parallel coordinates graphs. We demonstrate the applicability of the framework, named “Dynamics Visualisation based on Parallel Coordinates” (DYVIPAC), to a variety of signalling networks ranging in topological wirings and dynamic properties. The framework was proved useful in acquiring an integrated understanding of systems behaviour.

## Author Summary

In-depth understanding of molecular network dynamics in normal and pathological contexts is crucial in unravelling disease mechanisms and developing effective therapeutic approaches. Molecular networks are however overwhelmingly high-dimensional, often characterised by a large number of interconnected molecular nodes. Thus, network dynamics should be understood in a multi-dimensional setting, taking into consideration changes of as many network parameters as possible. Only then a more global and realiable picture of the systems dynamics could be obtained. Unfortunately, current ways to analyse and visualise systems dynamics of molecular networks are often limited to a very low number of network dimensions. Here, we develop an integrated framework termed “Dynamics Visualisation based on Parallel Coordinates” (DYVIPAC) which exploits the unique features of parallel coordinates graphs to visualise multi-dimensional systems dynamics. The framework generates innovative multi-dimensional plots in the form of parallel coordinates which are highly useful in gaining insights into how systems dynamics behave and are regulated.

## Introduction

Biochemical networks are highly complex and dynamic systems^1^,^2^. Their dynamic behaviours are often tightly regulated for normal cellular homeostatis, but also subject to changes by perturbations in pathological conditions, such as genetic alterations in the case of cancer^3^. The ability to obtain a global and accurate understanding of the dynamic behaviours of biochemical networks and their governing mechanisms in patho-physiological contexts are therefore of fundamental importance in biomedical research. Such systems understanding is key in the development of novel, effective network-based therapeutic strategies for diseases, that aim to target the network as a whole rather than a single isolated component^4^. Biochemical networks are however often high-dimensional and non-linear, consisting of a large number, often tens or hundreds, of interconnected components, that pose great challenges to the analysis of their behaviours.

Systems biology is an emerging paradigm that aims to address the complexity and interconnectedness of biochemical networks^5^,^6^,^7^. Network visualization and network-based mathematical modelling have become routinely used approaches in systems biology research. Inspired by graph theory, network visualization provides a graphical representation of the biological networks and a visual form of how the network components interact with each other, based on which useful insights regarding the topology, organisation and behaviour of the networks could be drawn^8^. As a result, a large repertoire of network visualization techniques and software tools have been developed for different purposes. Notable examples include visualization of the interactome^9^,^10^, large-scale network data^11^, gene regulatory^12^,^13^, signalling^14^,^15^ and metabolic networks^16^.

On the other hand, mathematical models provide a useful quantitative framework that enables systematic and rigorous analyses of the network dynamic properties. Importantly, model simulations generate predictions that can be used to formulate experimentally testable hypotheses^3^,^17^. Modelling and model-based analysis also play increasingly pivotal role in guiding the design and construction of synthetic biological networks^18^,^19^,^20^. Central to this approach are tools from dynamic systems theory including stability and bifurcation analyses, which are frequently used to dissect the dynamical properties of the networks and identify conditions that govern these dynamics^8^. As a result, the network (model) parameter space can be partitioned into sub-regions of distinct dynamic regimes, each presumably corresponds to a distinct biological phenotypes^17^,^21^,^22^. Such partitioning, in principle, would allow one to link model parameters to emergent network behaviours, thereby gaining important insights into the underlying regulatory mechanisms. However, the lack of effective visualisation techniques for high dimensional dynamics representation render current tools capable of only allowing dynamical partitioning on a very low dimensional space^17^,^21^,^22^. Consequently, what is often obtained is only a projection of the whole parameter space onto 2D planes called bifurcation plots. This limitation severely hampers our ability to gain a global and systems understanding of network behaviour.

**Figure 1:**
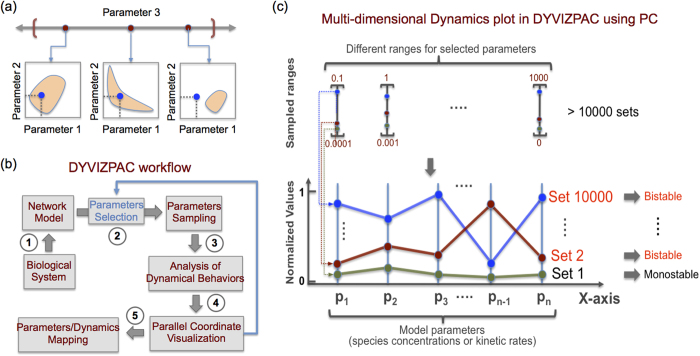
Description of the DYVIPAC framework. (**a**) Illustration of potential caveat when extrapolating global systems dynamics from observation of low-dimension (two-parameter) plots. (**b**) Workflow showing steps within DYVIPAC: explanation of each steps is described in the text. (**c**) Scheme illustrating the ensemble approach of dynamics analysis, characterisation and visualisation using PC plot in DYVIPAC. Typically, large sets (>10,000) of selected parameters (p_1_, p_2_,…, p_n_ where n ≥ 2) randomly sampled from specified ranges of values. The dynamic behavior of the modeled system at each set is assessed using linear stability analysis, then classified (e.g. bistable, monostable, oscillatory, etc.) and visualised using PC plot. On a PC plot, alues of each parameter p_1_, p_2_,…, p_n_ are represented on the corresponding (parallel) vertical axes and each line connecting the *n* parameter values represent a single parameter set. Note that all the sampling ranges are scaled to the [0,1] range to facilitate comparison and visualisation of all sampled sets on the same plot. The whole process can be repeated multiple times for different selection of parameters.

To further illustrate this conundrum with an example, let us consider a system that is capable of exhibiting sustained oscillation, which depend on at least three parameters. At different values of the third parameter within its range, a projection of the multi-dimensional oscillatory dynamics onto the plane of the first and second parameter could take markedly different forms (Fig. 1a). At the same values of these two parameters, the system may or may not oscillate depending on the value of the third parameter. This suggests that the interpretation drawn from a single bifurcation plot can yield misleading insights into the systems dynamics, if considered at higher dimension. To obtain a more complete picture of the systems dynamics, one would have to produce a large number of two-parameter plots at different set values of the third one. Importantly, the problem becomes even more cumbersome, when additional parameters are included into consideration, as bifurcation plots can hardly be visualised for more than three parameters. Moreover, limitations in gaining a global picture of systems dynamics strongly restrict our ability to correlate simultaneous changes in multiple biological parameters with systems behaviours, making it difficult to identify suitable parameters for desired network modulation in pathological contexts.

There is thus an unmet need to develop more efficient methods to analyse systems dynamics and visualise these dynamics in the context of multi-dimensional parameter space. In this paper, we address this need by presenting a computational and visualisation framework which ultilises the advantages provided by parallel coordinates graphs for displaying high dimensional data. This framework, termed “Dynamics Visualisation based on Parallel Coordinates” (DYVIPAC), is applied to a variety of biochemical networks and proved highly useful in gaining a more global understanding of the systems dynamics. We will first describe the principles of DYVIPAC in the next section. We then demonstrate the practicality of the framework by presenting detailed analyses of different networks ranging in wiring topologies and dynamic behaviours. Finally, we discuss potential path forward in future studies.

### Description of DYVIPAC

DYVIPAC is essentially an integrated analysis and visualisation framework for high-dimensional network dynamics. The major distinguishing feature of DYVIPAC lies in its ability to perform dynamical analysis of biochemical networks in multiple dimensions (parameters) simulaneously, and graphically represent these high dimensional dynamic data in a visually effective forms that facilitate biological insights. DYVIPAC comprises multiple steps involving both the analysis and visualisation of network dynamics, which are schematised in Fig. 1b and described in detail below.

DYVIPAC starts with a dynamic, kinetic model of a biological network of interest. The model can be newly developed or already existing, which uses ordinary differential equations (ODEs) that describe the time-dependent behaviour of the system. Following the selection of relevant model parameters, the number of which is not restricted, a parameter sampling algorithm is carried out that samples a large number of parameter sets over defined ranges in a random, unbiased manner from the uniform (or log-uniform) distributions. Thus, the number of dimensions that could be simultaneously analysed is only limited by the size of the model. The sampling range for each parameter typically spans physiologically plausible values, but can be relaxed from biological constraints to take any range for the exploration purposes. In the next step, an assessment of the network dynamics for each sampled parameter set is carried out based on local stability analysis^17^,^23^. This then enables a generic subdivision of the multi-parameter space into regions with distinct dynamic behaviours, such as monostability, oscillation, bistability, excitability, and others^17^. Subsequently, results from such dynamics classification can be visualised using parallel coordinates (PC). PC is a powerful visualisation technique used to visualise high-dimensional data in statistical sciences and data mining^24^,^25^. However, such visual representations have never been exploited in the context of systems dynamics. We apply it here to generate multi-parameter dynamics (MD) plots, as a key feature of DYVIPAC (Fig. 1c). As illustrated in Fig. 1c, an MD plot displays on the same graph multiple parameter sets which can correspond to different types of system dynamics. Each set consists of n parameter values, where n is the number of model parameters considered. The parameter values are presented on parallel, vertical bars while values belonging to the same set that generates particular system behaviour are connected by lines (Fig. 1c). MD plot thus provides a convenient and powerful way to represent high dimensional data on a plane.

The MD plot involves careful examination of the systems dynamics. For example, one can identify possible correlation and/or association between the parameters and types of dynamics, thus mapping changes in parameters to changes in systems behaviours. Importantly, as indicated in Fig. 1b, steps 2–4 can be iteratively performed for different selections of parameters. The MD plot represents how systems dynamics vary in a multi-dimensional parameter space, analogously to the 2D bifurcation diagrams, which consider only two parameters. This feature distinguishes DYVIPAC from other existing approaches.

In the following sections, we carry out detailed analysis using DYVIPAC for important types of biochemical networks encountered in cells. These include: (i) the negative feedback Goodwin system; (ii) the three-layer signalling cascade with negative feedback; (iii) the two-layer signalling cascade with positive feedback; (iv) the three-layer signalling cascade with mixed negative and positive feedback and (v) the multisite phosphorylation cycles system.

## Results

### The negative feedback Goodwin system

**Figure 2:**
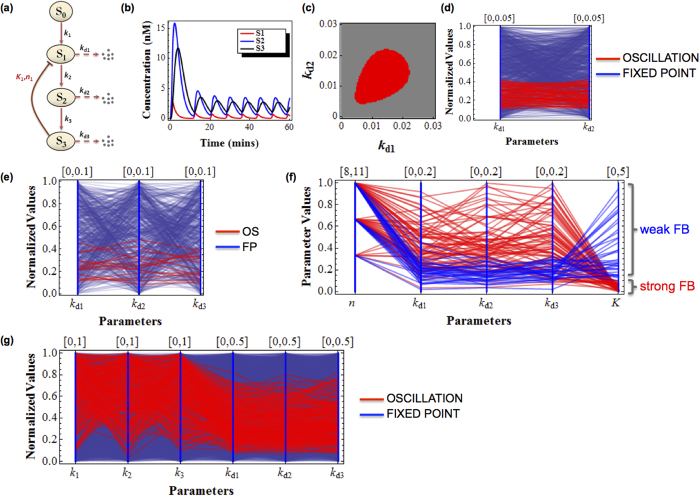
Multi-dimensional dynamics analysis of the Goodwin system with negative feedback. (**a**) Kinetic diagram of the Goodwin system. (**b**) Time-course simulation of sustained oscillations exhibited by the Goodwin system. (**c**) 2-D bifurcation diagram for parameters *k*_d1_ vs. *k*_d2_. Red indicates region of oscillation while grey indicates region of monostable fixed point. Parameters used are: *k*_1_ = *k*_2_ = *k*_3_ = 0.1 (nM s^−1^), *k*_d3_ = 0.01 (s^−1^), *n* = 9, *K* = 1 (nM). (**d**) PC-based bifurcation diagram for parameters *k*_d1_ and *k*_d2_. The remaining parameters values are as in (**c**). The sampling ranges for respective parameters are given on top of each vertical axis. All parameter values are normalised to the [0,1] interval. Note that the same notation applies hereafter for other PC plots. (**e**) PC-based bifurcation diagram for parameters *k*_d1_, *k*_d2_ and *k*_d3_. The remaining parameters values are as in (**c**). (**f**) PC-based bifurcation diagram for *k*_d1_, *k*_d2_, *k*_d3_ and *K*, *n*. The remaining parameters values are as in (**c**). Here, only the oscillation sets are displayed for clarity; the red lines indicate the sets with strong negative feedback (*K* < 0.1) while blue lines indicate those with weak feedback loop (*K* > 0.1). (**g**) PC-based bifurcation diagram for the rates of synthesis and degradation. The red lines indicate the sets giving oscillations while blue lines indicate monostable sets. The remaining parameters values are as in (**c**).

We start by considering a simple and well-known example of the negative feedback Goodwin model^26^. It was one of the earliest mathematical models formulated to describe genetic transcriptional repression^27^, and it was also adapted to model a variety of biochemical networks governed by negative feedback, particularly those capable of displaying oscillations^28^,^29^,^30^,^31^,^32^,^33^. In general, the model consists of three (or more) sequentially activating species, and the last species inhibits the first (Fig. 2a). The temporal dynamics of the Goodwin model with three species can be described by the following differential equations:


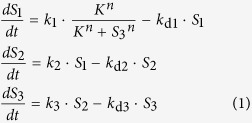


where *S*_i_, *k*_i_ and *k*_di_ (i = 1..3) denote the concentrations of species *S*_i_ and its synthesis and degradation rates, respectively. Negative feedback effect is modelled using the Hill kinetics^34^. For simplicity, the assumed constant input species *S*_0_ in Fig. 2a is lumped into rate constant *k*_1_. The synthesis and degradation rates are assumed to follow first-order kinetics. The half-saturating constant *K* describes the threshold level of the sigmoidal Hill function^34^,^35^, while the Hill coefficient *n* sig*n*ifies the level of nonlinearity.

Under proper parameter conditions, the Goodwin system can exhibit sustained oscillations (Fig. 2b). Figure 2c shows a conventional two-dimensional diagram of dynamics subdivision^36^ with regard to variation in the degradation rates k_d1_ and k_d2_ at certain values of the remaining model parameters. Oscillation is observed within a closed region of the plane (Fig. 2c). Figure 2d shows the equivalent output of a MD plot produced by DYVIPAC for k_d1_ and k_d2_. 10^3^ random sets were sampled over defined ranges for both parameters (parameter units are indicated in figure legends) before assessed for dynamics property. For convenience, we normalise all parameter ranges to between 0 and 1 to overcome possible differences in magnitude scale across model parameters. Note that MD plots without such normalisation are also available within DYVIPAC. Shown in Fig. 2d, the sets (lines) of {k_d1_, k_d2_} that give rise to sustained oscillation are highlighted in red and plotted together with the sets giving rise to monostable fixed-point, indicated in blue. Although distinct in presentation, similar to the conventional dynamics plot (Fig. 2c), the MD plot clearly shows that all the red lines are confined within restricted ranges on the k_d1_ and k_d2_ vertical axes, indicating that oscillation occurs only within these bounded ranges.

We now ask whether such constraint for oscillation still holds if other model parameters are allowed to change simultaneously. Answering this question is non-trivial with conventional approaches, as one would have to produce multiple 2D plots, each with a fixed parameterisation of the added parameters. This approach is often computationally costly and it is also challenging to manually extract meaningful insights from large ensemble of diagrams. DYVIPAC suggests a solution to this problem. When the third degradation rate k_d3_ is also allowed to vary, DYVIPAC analysis for {k_d1_, k_d2_, k_d3_} shows that oscillation occurs also only within confined ranges of all three parameters (Fig. 2e). Moreover, the almost horizontal orientation of the lines connecting data points of the oscillation sets (red) indicate that oscillation occurs at comparable values of three degradation rates, an observation in accordance with earlier analytical findings^36^,^37^.

To examine the effect of feedback strength on systems dynamics, we perform DYVIPAC for additional parameters, n and K_1_, subjecting 5 parametric dimensions to simultaneous changes. Here, the Hill coefficient n is sampled as integer values while, the threshold K is sampled over a continuous interval, ranging from very weak to very strong feedback inhibition. Of the 5 × 10^3^ random sets, Fig. 2f displays only those leading to oscillations for clarity, while the remaining monostable sets are hidden. Noticeably, more oscillation sets are detected as n increases. Particularly, no oscillation set is recorded for n < 8, suggesting this is a lower threshold for Hill coefficient to enable oscillations in the system, which actually agrees with previous analytical derivation^37^. In addition, the PC-based plot shows that most of the oscillation sets have low K, implying oscillation favours strong negative feedback as expected^37^.

Importantly, we no longer observe the oscillation-specific confined ranges for the degradation rates as seen in Fig. 2e when only these parameters are varied. This suggests that varying the feedback strength relaxes the above constraints on degradation rates for oscillation, an observation not available with conventional approaches. A closer interrogation of the plot gives additional insights. By introducing an empirical cut-off value for K, we find high Hill function threshold (high K) is exclusively associated with low degradation rates (blue lines, Fig. 2f), whereas low threshold (low K) shows no such feature (red lines, Fig. 2f). This implies that a weak negative feedback loop necessitates slow degradation rates to enable oscillation^33^. We further perform DYVIPAC for all the turnover-related kinetic rates (Fig. 2g). Oscillation is more likely at higher synthesis rates whereas equally low synthesis rates do not result in oscillation regardless of the degradation rate values. Interestingly, simultaneous changes in the synthesis rates do not relax the constraints confining the ranges of the degradation rates (red lines, Fig. 2g).

### The oscillatory MAPK cascade with negative feedback

**Figure 3:**
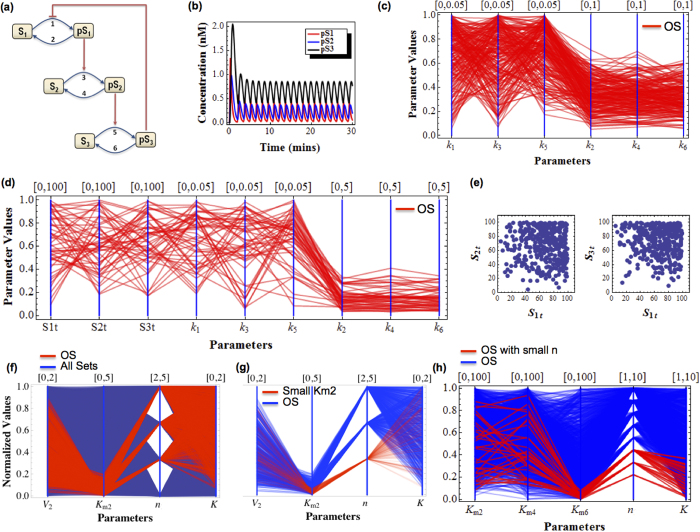
Multi-dimensional dynamical analysis of the 3-layer MAPK cascade with negative feedback. (**a**) Schematic diagram (**b**) Timecourse of sustained oscillations (**c–e**) Multi-dimensional bifurcation analysis of the cascade when (de)phosphorylation reactions follow first-order kinetics for: (**c**) the rate constants (**d**) the rate constants and species abundances (**e**) scatter plots of relevant quantities. (**f–h**) Multi-dimensional bifurcation analysis of the cascade when (de)phosphorylation reactions follow saturating kinetics (MM): (**f**) oscillations sets (red) superimposed on all generated sets (blue) when only reaction 2 is described by MM kinetics (**g**) oscillations sets as in (**j**) but those with n = 3 is highlighted in red. (**h**) oscillations sets (blue) when all the reaction 2,4,6 are described by MM kinetics with those having low n highlighted in red.

Signal transduction via cascades of reversible phosphorylation cycles is a hallmark of cell signalling. The highly conserved and extensively studied mitogen-activated protein kinase (MAPK) signalling cascade is known to play important physiological roles, including proliferation, differentiation and apoptosis^3^,^38^. MAPK cascade consists of multiple levels of (de)phosphorylation cycle catalysed by a kinase of the preceding level and a phosphatase of the given level (Fig. 3a). In addition, multiple negative and/or positive feedbacks confer the cascade with potentially complex dynamic behaviours^2^. In this section, we examine the three-tier MAPK cascade controlled by a negative feedback loop (Fig. 3a), exemplified by the MAPK ERK pathway^3^,^39^. Sustained oscillation was predicted for this system under physiological conditions^39^ (shown in Fig. 3b) and confirmed by recent experimental reports^40^,^41^. Although condition underlying oscillation in the system has been examined to some extent^39^, a multi-dimensional characterization is lacking. Here, we employ DYVIPAC to fill in this knowledge gap. Following the schematic diagram given in Fig. 3a, the reaction rates are written as follows,


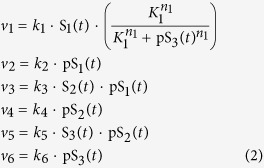


The bracket term in *v*_1_ describes negative feedback effect using the Hill kinetics. Parameters *k*_1–6_ represent the catalytic rate constants of the respective reactions using first-order kinetic law. Our goal here is to first analyse the reaction rates as bimolecular interactions and then later analyse the influence of Michaelis-Menten nonlinearity. We have the following conservation laws:


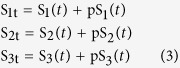


where S_1t_ , S_2t_ and S_3t_ are the total concentrations of species S_1_, S_2_, S_3_. Substitute these conservations into the eqn. (2), the MAPK system is governed by the following ODEs:


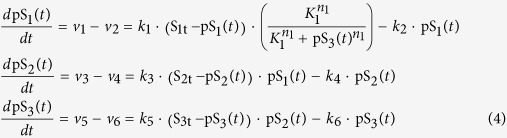


First, we implement DYVIPAC analysis for 6 kinetic rate parameters controlling (de)phosphorylation cycles, k_1_-k_6_. Among 10^4^ sampled sets, the network exhibits either sustained oscillation or fixed point monostability. Figure 3c displays only those that give rise to oscillations. While parameter sets leading to monostable behaviour do not exhibit any characteristic feature, observing the oscillation sets reveals that the corresponding dephosphorylation rates, k_2_, k_4_ and k_6_, are necessarily confined within strict, comparable ranges (Fig. 3c). Notably, these rate constants remain to be in the similar confined range even when we varied S_1t_, S_2t_ and S_3t_, thus extending the analysis to 9 parameters (Fig. 3d). Similar to the Goodwin system, comparable dephosphorylation rates are more likely to drive oscillation, indicated by the nearly horizontal lines connecting the oscillation sets, while phosphorylation rates should be sufficiently fast. Interestingly, as seen in Fig. 3c,d oscillation sets having slow phosphorylation rate in one layer tend to associate with fast phosphorylation rate in the next layer, suggesting that aggregated signalling flux through the cascade should be fast enough to allow oscillation to realise. We also found that none of the abundance levels of S_1_, S_2_ and S_3_ are too low when oscillations are observed (Fig. 3d). This is further supported when we projected the multi-dimensional oscillation sets in Fig. 3d onto a 2D plane using scattered plots for the species abundances (Fig. 3e), indicating high, comparable abundances are favourable for oscillation. It is worth noting that despite their differences, the conditions governing oscillations for the rates of (de)phosphorylation in the MAPK cascade resembles that governing the synthesis and degradation rates in the simple Goodwin system.

Conducting DYVIPAC for the feedback parameters showed that low threshold (low K_1_) and high nonlinearity (n_1_) enhance oscillations as expected, while weak feedback requires slow dephosphorylation rates to enable oscillation. Interestingly, we also found that n_1_ has a strict lower bound (which is 8) confirming that nonlinearity is conditional for sustained oscillatory dynamics.

This led us to question whether the requirement for nonlinearity could be compensated by different parts of the network. To examine this, we introduced nonlinearity to other network reactions, by assuming the (de)phosphorylation now follow Michaelis-Menten (MM) instead of the linear first-order kinetics (ODEs given in SI). We first described only reaction 2 using MM kinetics (Fig. 3a), where the MM constant K_m2_ represents the nonlinearity level. Multi-dimensional analysis for 4 parameters involving two sources of nonlinearity—the dephosphorylation reaction (V_2_, K_m2_) and the negative feedback (n_1_, K_1_) – showed that oscillation now become possible at much lower n_1_ (as low as 3), owing to the shared nonlinearity brought about by low K_m2_ (red lines, Fig. 3f) which conditions reaction 2 to operate in a strong saturating regime. In addition, by highlighting oscillation sets with different values for n_1_ using different colours we observed that low n_1_ exclusively correlates with low K_m2_ while increasing n_1_ expands the oscillatory range of K_m2_ and relaxes the saturation constraint (Fig. 3g—may move to SI). Taken together, these results show that increased nonlinearity provided by low K_m2_ can compensate for the reduced nonlinearity brought about by the negative feedback with a low n_1_.

Similar observation was made when dephosphorylation reactions 4 or 6 were assumed to follow the MM kinetics. Interestingly, in all cases out of 10^4^ randomly sampled sets having n_1_ = 2, no single set can oscillate; while tallying sets with n_1_ > 2 shows that higher n_1_ promotes oscillations, as expected. Next, we introduced nonlinearity to all the cascade levels by modelling all 3 dephosphorylation reactions using MM kinetics. Unexpectedly, DYVIPAC analysis for the three K_ms_ parameters and the feedback parameters demonstrated that oscillation at low n_1_ also requires low K_m6_ but not low K_m2_ or K_m4_ (red lines, Fig. 3h). This suggests that saturation of the last dephosphorylation reaction is critical in enabling oscillation at low n_1_. Raising n_1_ extends the range of K_m6_ possible for oscillation. Combined, these results indicate the nonlinearity requirement for oscillation can indeed be shared among distributed parts of this network given that sufficient aggregated network-wide nonlinearity level is met.

### A bistable signalling cascade with positive feedback

**Figure 4:**
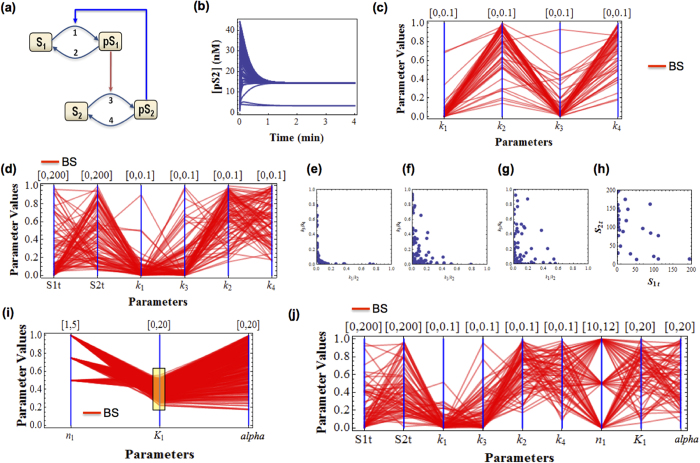
Multi-dimensional dynamical analysis of the 2-layer MAPK cascade with positive feedback. (**a**) Schematic diagram (**b**) Illustration of bistable response displayed by the cascade: depending on the starting point (i.e. initial condition), the system ends up in either one of two steady states. Here, the system is initialised differently by random initialisation of the concentrations of pS_1_ and pS_2_ given that the total S_1_ and S_2_ levels are fixed. Parameter values used: S_1t_ = 30, S_2t_ = 45, *k*_1_ = 0.002, *k*_2_ = 0.4, *k*_3_ = 0.005, *k*_4_ = 0.04, *n* = 10, *K* = 7, *α* = 5. (**c,d,h,i**) Multi-dimensional bifurcation analysis of the cascade for: (**c**) the kinetic rate constants (**d**) the rate constants and species abundances (**h**) the feedback-related parameters (here the bounded range of *K* is highlighted) and (**i**) all model parameters.(**e–g**) Scatter plots of the relevant quantities: k_1_/k_2_ vs. k_3_/k_4_ plotted for the bistable parameter sets in panel (**c**,**d**) and (**h**) respectively. (**k**) Scatter plots of the relevant quantities: S_1_ vs. S_2_ for the bistable parameter sets in panel (**d**).

In this section, we consider the two-tier cascade with positive feedback (Fig. 4a), focusing on the regulation of positive feedback-induced bistability by different parameters using DYVIPAC. Following the network scheme in Fig. 4a, the reaction rates are described as:


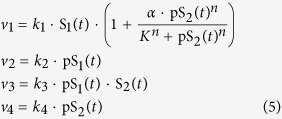


The term in *v*_1_ that depends on S_2_ describes the positive feedback effect from pS_2_ to S_1_, modelled using the Hill function. Parameters α, n and K represent the feedback strength, nonlinearity level and feedback activation threshold, respectively. *k*_1_–*k*_4_ are the kinetic rate constants of the respective reactions. In addition, the following conservation laws apply:


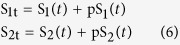


where S_1t_ and S_2t_ are the species abudances. Substitute these laws into the reactions rates, we derived the ODEs for the cascade as follows.


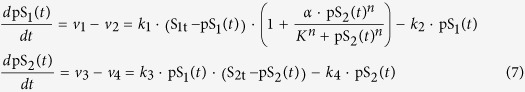


Positive feedback in biochemical networks can bring about switch-like and bistable responses^5^,^17^,^42^. Under appropriate parameterisation, the cascade in Fig. 4a exhibits bistability as shown in Fig. 4b where the phosphorylated S_2_ settles in either one of two distinct steady states depending on its initial starting level. On the other hand, oscillation is not feasible due to the lack of negative feedback, making bistability and monostable fixed-point the primary dynamics displayed by the cascade.

Running DYVIPAC with 10^3^ sets for all the kinetic rates *k*_1–4_ clearly showed that bistability occurs premarily at slow phosphorylation (low k_1_, k_3_) relative to the dephosphorylation steps (high k_2_, k_4_) (Fig. 4c displays only bistable sets). Scatter plot of k_1_/k_3_ versus k_2_/k_4_ for only the bistable sets further supports this PC plot finding, as the majority of parameter values are confined to the proximity of the axes (Fig. 4e). Moreover, there appears to be a low threshold for the dephosphorylation rates below which bistability does not occur, suggesting that fast dephosphorylation is critical for bistability (Fig. 4c). Importantly, extending the analysis to 6 parameters by additionally varying the species abundances reveals similar patterns for the kinetic constants that generate bistability (Fig. 4d), despite the constraint on low k_1_/k_3_ and k_2_/k_4_ is slightly relaxed (Fig. 4f). A distinct asymmetric correlation was detected by this multi-dimensional analysis between total S_1_ and S_2_, where low S_1t_ strongly associates with high S_2t_ and vice versa (Fig. 4d,h).

We next ask how the positive feedback regulates bistability. A 3-parameter analysis involving only the feedback related parameters showed that bistability exists under several stringent criteria (Fig. 4i). First, the feedback loop must possess sufficient level of nonlinearity as no bistable sets were recorded for n < 3. Second, the feedback must be strong enough indicated by a lower bound for parameter α. Third, the feedback activation threshold (K_1_) is required to reside within a confined range. While the former two conditions are preserved when we performed DYVIPAC for all model parameters, the third condition is not preserved as K_1_ occupies a large range of values (Fig. 4j). Surprisingly, the patterns of the species abundances and kinetic rates still hold under this comprehensive mode displayed by DYVIPAC.

### A signalling cascade with mixed feedback regulations

**Figure 5:**
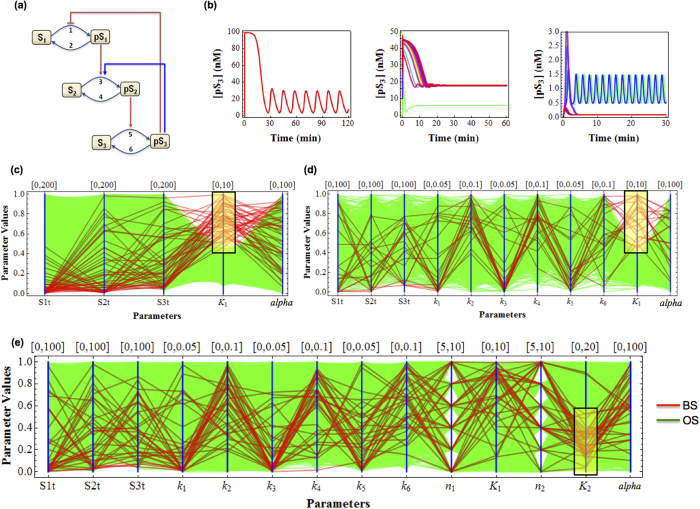
Multi-dimensional dynamical analysis of the mixed-feedback, 3-layer MAPK cascade. (**a**) Schematic diagram of the mixed feedback cascade. (**b**) Three types of dynamics can occur in the system: oscillations, bistable fixed-points, or bistability with mixed oscillatory and fixed-point steady state. Here, the system is initialised differently by random initialisation of the concentrations of pS_1_, pS_2_ and pS_3_ given that the total S_1_, S_2_ and S_3_ levels are fixed. Parameter values used for oscillation S_1t_ = 100, S_2t_ = 100, S_3t_ = 100, *k*_1_ = 0.1, *k*_2_ = 0.01, *k*_3_ = 0.01, *k*_4_ = 0.01, *k*_5_ = 0.01, *k*_6_ = 0.01, *n*_1_ = 10, *K*_1_ = 1, *n*_2_ = 15, *K*_2_ = 8, *α* = 10; for bistable fixed point: S_1t_ = 0.22, S_2t_ = 10, S_3t_ = 53, *k*_1_ = 0.0012, *k*_2_ = 0.006, *k*_3_ = 0.049, *k*_4_ = 0.084, *k*_5_ = 0.043, *k*_6_ = 0.066, *n*_1_ = 5, *K*_1_ = 9.5, *n*_2_ = 10, *K*_2_ = 15, *α* = 95 ; and for mixed bistability: S_1t_ = 20, S_2t_ = 50, S_3t_ = 30, *k*_1_ = 0.001, *k*_2_ = 0.08, *k*_3_ = 0.001, *k*_4_ = 0.08, *k*_5_ = 0.001, *k*_6_ = 0.05, *n*_1_ = 10, *K*_1_ = 0.66, *n*_2_ = 5, *K*_2_ = 0.8, *α* = 96. (**c–e**) Multi-dimensional bifurcation analysis of the cascade for: (**c**) the species abundances and feedback strength (**d**) the species abundances, the rate constants and feedback strength and (**e**) all parameters. The yellow boxes are for highlighting purpose. Bistable (BS) and oscillation (OS) sets are indicated in red and green, respectively.

To illustrate the applicability of DYVIPAC to a system in which both oscillation and bistability may occur, we consider the three-tier signalling cascade with mixed positive and negative feedback regulation, as illustrated in Fig. 5a. The reaction rates are described as follows.


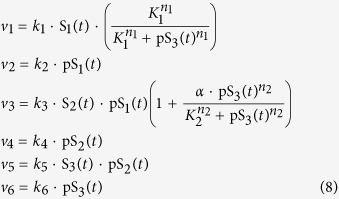


The terms in brackets in *v*_1_ and *v*_3_ present the negative and positive feedback effects, respectively, as described in the previous sections. Substituting the species conservation laws (Eq. 2a) into the rate equations, we arrive at the following ODE system involving the phosphorylated form of S_1_, S_2_, and S_3_:


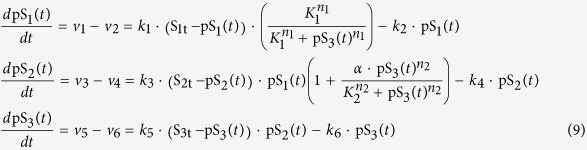


The mixed dynamics including oscillation and bistable response are displayed in the cascade under appropriate parameter values (Fig. 5b). To interrogate the contributing roles of the opposing feedback loops in the regulation of the cascade dynamics, we performed DYVIPAC for various combinations of model parameters. First, we looked at the species abundances and parameters governing the feedbacks’ strength (K_1_ and α). Out of 10^4^ random generated sets for DYVIPAC over wide ranges, about 90%, 9% and 1% are monostable, oscillatory or bistable, respectively. These counts suggest that oscillation is much more likely than bistability in the mixed cascade.

Importantly, looking only at the sets leading to oscillation or bistability (Fig. 5c), we observed a cutoff value of K_1_, which represents the activation threshold of the negative feedback. Sets resulting in bistability appear to exclusively occupy the upper part while oscillatiory sets fill the lower part of the sampled range. Such a sharp cutoff was also visible even when we performed the analysis taking into account all the kinetic parameters, increasing the total dimensions being examined to 11 (Fig. 5d). Strength of the negative feedback appears therefore not only a strong predictor of oscillation, but also of bistability. Strong negative feedback (low K_1_) is strictly required for oscillation whereas this prevents the occurrence of bistability. Perturbing the negative feedback hence presents the most straightforward and probable way to shift systems behaviour from oscillatory to bistable behaviour. On the other hand, increasing the positive feedback or other parameters solely may not be as an effective strategy (Fig. 5d).

To further assess if and how the presence of the positive feedback loop influences the emergence of oscillation, we calculated from nearly 5000 oscillatory sets returned from DYVIPAC those having different levels of the parameter α. Interestingly, the number of sets with very high (top 5% of the sampled range) and very low α (bottom 5%) are comparable, accounting for about 10% of the total sets in both cases. We carried out similar tallying for the remaining parameters controlling the positive feedback, K_2_ and n_2_. Within the oscillatory sets, we observed that low K_2_ is strongly associated with low K_1_. Moreover, the number of sets having high K_2_ (14%) is significantly higher than those having low K_2_ (3%). These results indicate that a positive feedback loop with increased activation threshold (K_2_), rather than increased strength (α), would promote oscillation by allowing it to realise over a much wider strength of the negative feedback. Similar analysis for n_2_ showed that higher Hill coefficient for the positive loop also enhances oscillation (not shown). Taken together, the presence of positive feedback confers oscillation with enhanced robustness, a result in line with previous studies^43^. On a practical note, one should however be cautious when interpreting the so-called strength of a positive feedback, which we think should be a combined assessment of both parameters K_2_ and α.

In addition, to visualize the global picture of the systems behaviour, we ran DYVIPAC for all model parameters (14 in total, Fig. 5e), which is practically impossible with conventional methods. We observed consistent trends regarding the feedback parameters as seen above, strengthening our confidence that the conclusions drawn from the PC plots refect a faithful multi-dimensional dynamics assessment of the system. Moreover, we also detected similar correlation patterns between the kinetic rates within the bistable sets, as presented for the bistable two-layer cascade , where slow dephosphorylation rates (k_1,_k_3,_k_5_) tend to be associated with fast phosphorylation rates (k_2,_k_4,_k_6_). These high dephosphorylation relative to phosphorylation rates within each cycle of the cascade remain a requirement for bistability under the mixed feedback regulation.

### System of multisite phosphorylation cycles

**Figure 6:**
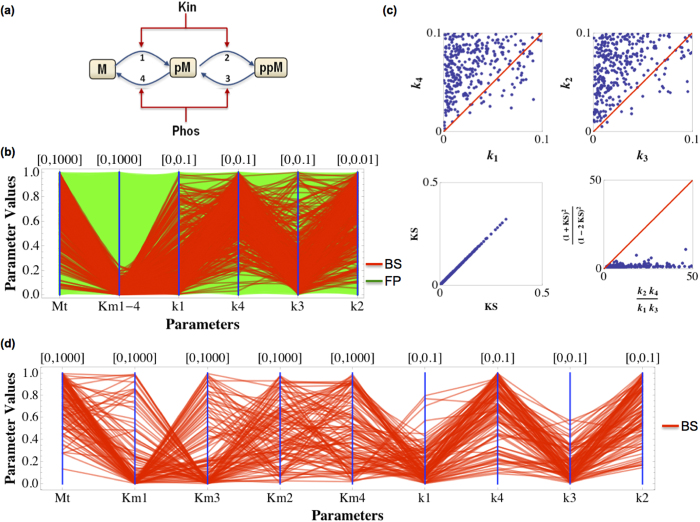
Multi-dimensional dynamical analysis of the multisite phosphorylation system (**a**) Schematic diagram of the system. (**b**) Multi-dimensional bifurcation analysis for M abundance, the K_m_s constants and kinetic parameters k_1–4_. Two types of dynamics can occur in the system: bistability (red) and fixed-point steady state (greed). (**c**) Scatter plots of relevant expressions of the same data in panel (**b**). (**d**) Multi-dimensional bifurcation analysis of the system for all model parameters except [Kin] and [Phos]. The reference parameter values used are: Parameter values used for oscillation M_t_ = 100, K_in_ = 10, Phos = 10, *K*_m1_ = *K*_m2_ *=* *K*_m3_ *=* *K*_m4_ = 100, *k*_1_ = 0.01, *k*_2_ = 1, *k*_3_ = 0.084, *k*_4_ = 0.06.

As the final illustrating example, we re-examine a multisite phosphorylation cycle where bistability can occur without explicit positive feedback^42^ using the DYVIPAC multi-dimensional analysis. The kinetic scheme of this system is shown in Fig. 6a. Step 1 and 2 are modified by a common kinase (Kin) while step 3 and 4 are modified by a common phosphatase (Phos). We have previously analytically analysed the kinetic requirements for bistability in this system under simplifying assumptions^42^,^44^, here we demonstrate that DYVIPAC can be used to further characterize the occurrence of bistability in the system when simplifying assumptions are relaxed.

Under the quasi steady-state assumption, the rate equations (v_i_) have the following form (see^44^ for derivation):


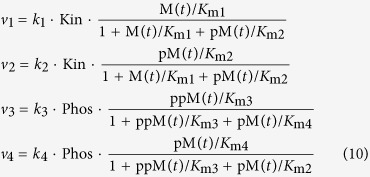


where M, pM and ppM denote the concentrations of the unphosphorylated, singly and doubly phosphorylated forms of the species M. *K*_mi_ are the Michaelis constants and *k*_i_ are the kinetic rate constant of step *i* for *i* = 1..4. Applying the conservation law:


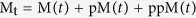


where M_t_ is the total abundance of M, the differential equations that govern the time evolution of the system shown in Fig. 6a can be presented as,


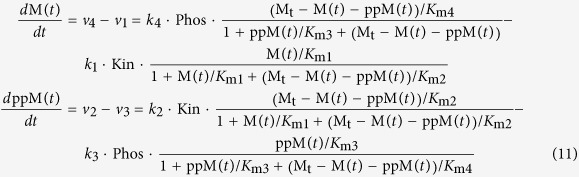


For simplicity, we first assumed that the Michaelis-Menten constant *K*_m_s are equal for all reaction steps 1–4 (*K*_m1–4_). We then subjected 9 parameters including the abundance of M (M_t_), *K*_m1–4_ and the kinetic rate *k*_1–4_ to DYVIPAC analysis. A total of 10^5^ parameter sets were randomly generated as input and checked for possible dynamics. Monostable fixed point and bistability are the only two types of dynamics detected, among which only about 350 are bistable sets accounting for just 0.3% of the total sets (Fig. 6b). Shuffling the axes on the PC plot such that *k*_1_, *k*_4_ and *k*_3_, *k*_2_ are next to each other revealed visible patterns among the bistable sets: low *k*_1_, *k*_3_ are associated with high *k*_4_ and *k*_2_, respectively (Fig. 6b, red lines). Scatter plots of *k*_1_ vs. *k*_4_ and *k*_3_ vs. *k*_2_ further show that most of the bistable sets have *k*_1_ < *k*_4_ and *k*_3_ < *k*_2_ (Fig. 6c). We also observed a strong and consistent positive correlation between *K*_m1–4_ and M_t_, suggesting the existence of some constraint on these parameters. Our previous analysis under the simplifying assumption of equal *K*_m_s arrived at an analytical constraint essentially stating that bistability exists when: *k*_2_*k*_4_/*k*_1_*k*_3_ > (1 + β)^2^/(1–2β)^2^ and β < 1/2 where β = *K*_m_/M_t_ is the common Michaelis-Menten constant normalised by M abundance^44^. We thus checked if the bistable sets numerically returned in Fig. 6b would fit the analytical conditions. The lower panels of Fig. 6c show this is indeed the case, confirming the previously derived analytical condition. Interestingly when we fixed the value of *K*_m1–4_ and only varied M_t_ and the kinetic rates, the PC as well as the scatter plots revealed a more strict parametric relation among the bistable sets: *k*_1_ < *k*_4_ and *k*_3_ < *k*_2_. Taken together, these combined results indicate that under the equal *K*_m_s assumption, the system is most likely to display bistability if M’s abundance strongly exceeds the *K*_m_ level and that the relative rate of dephosphorylation over phosphorylation of the first cycle is slow whereas that of the second cycle is fast.

Although the condition for bistable dynamics is not tractable analytically, when the *K*_m_s are different, we further explore the parameter ranges using DYVIPAC. We generated 10^5^ random sets with simultaneous variation of all model parameters except the concentrations of Kin and Phos (Fig. 6d) as inputs for the DYVIPAC analysis. We observed 111 bistable sets, accounting for 0.1% of the total sets. Interestingly, these bistable sets all satisfy *k*_2_*k*_4_/*k*_1_*k*_3_ > 1 (Fig. S4a), suggesting this necessary condition applies even under the case of different *K*_m_s. Closer examination of the bistable sets revealed that those with high *K*_m1_ (or *K*_m3_) always associate with low *K*_m3_ (or *K*_m1_), as displayed in Fig. S6b,c. However, low *K*_m1_ (or *K*_m3_) can associate with all possible values of *K*_m3_ (or *K*_m1_). No apparent correlation were detected for *K*_m2_ and *K*_m4_ as values spanning their whole range were seen associated with both low and high *K*_m1_ or *K*_m3_. Taken together, these results provide new insight indicating that at least the phosphorylation of M or the dephosphorylation of ppM should operate in a near saturating regime (low *K*_m_) for bistability to display. However, no such restriction is required for the (de)phosphorylation steps of the intermediate phosphorylated species pM.

Finally, we ran DYVIPAC for all the model parameters including the concentrations of the modifying kinase and phosphatase. Under this most general case, the observed correlation patterns among model parameters detected above still exhibit, suggesting that the most likely conditions for bistable response are: the phosphorylation of M and dephosphorylation of ppM operate in a strong saturating regime, and the phosphorylation compared to dephosphorylation of the first cycle is slow whereas that of the second cycle is exceedingly fast.

## Discussion

### Folded PC plot for direct parameter-to-parameter correlation

**Figure 7:**
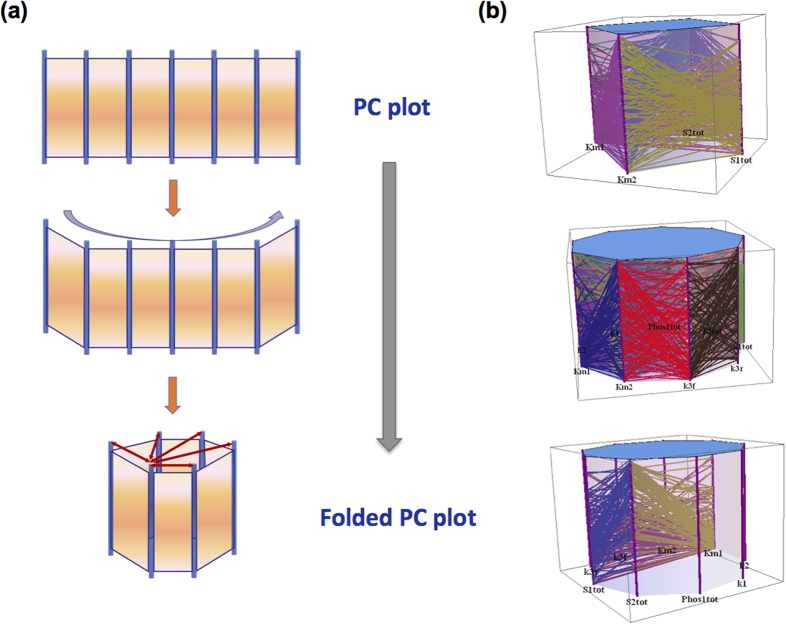
Principle of the folded PC plot (**a**) Illustration of the folded PC plot formed by folding the normal PC plot. (**b**) Illustrative folded PC plot for 4 (upper panel), 9 (middle & lower panels) parameters.

The standard representation of the PC-based MD plot, illustrated extensively through the above examples using a series of vertical axes, has helped us gain insights into the systems behavior by detection of multiple parameter patterns that underlie a particular dynamics type. Nevertheless, such representation faces a practical limitation when one wants to quickly correlate any pair of parameters without having to rearrange the vertical axes’ positions in order to bring the pair of axes next to each other (Fig. 7a). Although DYVIPAC allows for the ability to interactively swap axes to address the issue, we aimed to design a complementary PC representation where swapping is not even required. This can be done by folding the standard PC plot in 3D to form a novel type of plot we name “folded PC plot” that allows all parametric axes to be connected directly to each other without swapping, an idea motivated by observing the traditional Chinese fold-screen furniture (i.e. ping-feng in Chinese, Fig. 7a). Using the folded PC plot which can flexibly display and/or hide different surfaces connecting pairs of axes, one can directly correlate one parameter to any other parameter in a convenient manner (Fig. 7b). Moreover, we envision that this novel plotting technique would be of benefit in a wider context for any data visualised using parallel coordinates.

### Assessment of dynamics: local stability versus continuation analysis

Evaluation of systems dynamics within the DYVIPAC framework is currently based on local stability analysis of the systems steady state solutions at a specific parameterisation, where the eigenvalues of the corresponding Jacobian matrix are numerically calculated and systematically classified^17^. For example, consider the mixed-feedback MAPK cascade which has either one or three steady state solutions for the considered ranges of parameters. When a unique steady state exists, the sign of the largest real part of the eigenvalue associated with that solution allows us to classify that steady state as stable or unstable, indicating potential presence of sustained oscillations. On the other hand, when three steady states coexist, two stable and one unstable solution indicate a bistable dynamical behavior. The borders separating these different regions correspond to different kind of transitions. The points belonging to the borders separating the cases when one or three solutions exist are called saddle nodes. The points belonging to the borders where any of the steady states lose its stability (the largest negative real part of the eigenvalue becomes positive) are called the Hopf bifurcations. In principle, other possible dynamics can be detected based on local stability analysis such as excitable behaviour (two unstable and one stable steady states)^17^,^45^.

Although we constrained DYVIPAC to local stability analysis as determined by the eigenvalues, it is important to note that a selected parameter set may correspond to a complex bifurcation, which may not be evident from the eigenvalue analysis. Several existing programs, most notably AUTO and XPPAUT^46^, can conduct such bifurcation analysis using sophisticated continuation algorithms but unfortunately is only restricted to two parameters at most, i.e. generating only a two-dimensional picture of the systems dynamics. DYVIPAC trade-offs accuracy for visibility and speed, providing efficient and convenient visualisation of generic subdivision of the “global” multiparameter space, which in many cases, may be more useful in guiding experimental efforts. Moreover, with the development of continuation methods for multiple parameters in future studies, one can imagine exploiting the PC visualisation component of DYVIPAC to accommodate results coming from such methods when available, which would offer a formal way to represent true multi-parameter bifurcation diagrams for systems dynamics.

### Parameter sampling and sampling algorithms

The type of distribution and parameter sampling algorithm used for DYVIPAC can influence the outputs. Since the primary aim of parameter sampling within DYVIPAC is to exhaustively explore the parameter space, the default distributions are uniform or log-uniform if the parameter ranges span several orders of magnitude. This is in contrast to log-normal distribution typically used for sampling protein abundances for simulations of cell-to-cell variabilities^47^. We employ Monte Carlo (MC) as the default method of sampling because of its simplicity and efficiency shown while applying the framework to analysing example networks. However, due to the random nature of the MC sampling, we risk missing some regions of the parameter space, particularly when the number of dimensions is increased and the number of sampled sets is not sufficiently large. To overcome such risk, we also implemented sampling using the Latin Hypercube Sampling (LHC) method^48^, which is designed to ensure a more complete coverage of the parameter space by dividing each parameter range into multiple segments and sampling within each segments before combining the sampled values into sets. For example using 5 segments for sampling 5 parameters would in principle require only about 3000 sets (5^5^) for a good exploration of the 5-parameter space. In our experience, which method and/or the number of segments used should be a flexible decision by users depending on specific context. LHC is generally more advantageous when the number of considered dimensions is greater than 10, while MC can be sufficient at lower dimensions.

### DYVIPAC implementation, usage and performance

**Figure 8:**
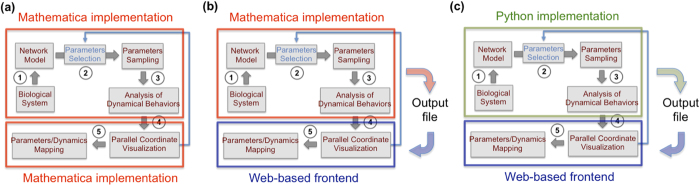
Current complementary implementations of DYVIPAC. (**a**) Full implementation in Mathematica. (**b**) Dynamical analysis implementation in Mathematica producing output file that could then be rendered as MD plots using a web-based frontend for parallel coordinate visualisation. (**c**) Dynamical analysis implementation in Python producing output file that could be rendered as MD plots using a web-based frontend for parallel coordinates visualisation (see text for detailed discussion).

DYVIPAC was designed as a modular pipeline composing of related but separate steps, it thus could be utilised in several ways. We have implemented DYVIPAC in three different flavours that allow the users to capitalise on different implementation platforms for their needs (Fig. 8).

Firstly, DYVIPAC was fully implemented in Mathematica, one of the most common and powerful computational platforms for systems biology research^49^. The Mathematica code of the framework and the example models analysed in this paper are provided with this manuscript as Supplementary Information. Thus, the users could conduct additional analysis of the tested models, or fresh analysis of their own models by inputing the models in the form of an ODE system. In this way, all DYVIPAC’s steps including the dynamical analysis and MD plot rendering are performed within Mathematica (Fig. 8a). Alternatively, the users can conduct the dynamical analysis steps in Mathematica but instead displaying the corresponding MD plots in a separate visualisation frontend which gives more flexibility and interactive features. To this end, we implemented a web-based visualisation tool for parallel coordinate, which takes data files as output from the dynamical analysis and renders the MD plots (Fig. 8b). This web-based frontend provides users with state-of-the-art features in parallel coordinate visualisation including brushing (highlight of a particular part of one or more vertical axes), axes reordering, axis inverting and removal, which would further aid the users in interrogation of the MD plots for in-depth biological insights. This tool and detailed instruction can be accessed from http://137.43.208.102:7070/SynSig.

To enable parallel processing and allow users to utilise the power of high-performance computing, we have also implemented the dynamical analysis steps of DYVIPAC in Python using the API of the libRoadRunner 1.3.1 library (libroadrunner.org)^50^ (Fig. 8c). Importantly, this implementation allows the users to load models in Systems Biology Markup Language (SBML) format, the most widely used standard for representing dynamic biochemical models. As in the Mathematica implementation, output of the dynamical analysis using the Python implementation can be saved as data files, which are then visualised using our web-based parallel coordinate tool described above (Fig. 8c). The Python implementation allows DYVIPAC to run on multi-CPU clusters, which could substantially reduce the computational time associated with dynamical analysis with large parameter sampling size. An online instruction of this implementation is given at https://bitbucket.org/andreadega/dyvipac-python. In summary, depending on the need and infrastructure at hand, the users could carry out the dynamical analysis components using the Mathematica or Python implementations, and render the parallel coordinate based MD plots using the web-based visualization tool or in Mathematica.

It is noted that the most computationally time-consuming part of DYVIPAC is the model’s stability analysis steps while the rendering of the parallel coordinate plots using our web-based frontend is quick. To provide a general guide on the computational time of DYVIPAC under different implementations, we report the running time of DYVIPAC on two example models analysed in the paper for various specifications (Table 1). As could be seen in Table 1, DYVIPAC’s performance depends directly and proportionally on the number of sampled parameter sets, which significantly decreased as the framework was run using multiple processing cores (from 1 to 8 cores using the Python implementation). The running time in Mathematica is slightly slower compared to that of the Python implementation for the tested cases. In addition, the number of parameters being simultaneously analysed also seemed to affect the running time. It however should be noted that Table 1 only serves as a rough guide since the actual running time is strongly determined by the complexity, nonlinearity and stiffness of the dynamic models.

## Concluding Remarks

Visualisation plays a significant role in interpretation of biological findings. Biologists can visualise molecular interactions with pathway maps, while modellers present biochemical networks via graphical schemes and interactive simulations. A wealth of multi-variate -omics and imaging data produced by high-throughput techniques further places visualisation as a fundamental tool in the intepretation and translation of these data into biological knowledge. A key aspect of understanding how cells behave in pathophysiological contexts is the ability to comprehend the dynamical properties of molecular networks in their multi-dimensional settings. DYVIPAC was aimed at addressing the limitations imposed by existing methods, integrating the analysis and visualisation of dynamics of high-dimension biochemical networks. The framework generates multi-dimensional plots in the form of parallel coordinates which help in detecting patterns between multiple model parameters that characterise particular types of dynamical behaviours. Applications of DYVIPAC to networks ranging in dynamical complexity and topologies have demonstrated the robustness of this approach. Furthermore, DYVIPAC can be useful in guiding the design and parameter tuning of molecular networks for a desired behaviour, a task required in the emerging field of synthetic biology.

## Additional Information

**How to cite this article**: Nguyen, L. K. *et al.* DYVIPAC: an integrated analysis and visualisation framework to probe multi-dimensional biological networks. *Sci. Rep.* **5**, 12569; doi: 10.1038/srep12569 (2015).

## Supplementary Material

Supplementary Information

## Figures and Tables

**Table 1:** Performance of DYVIPAC.

**DIVIPAC Specification**				**Running Time**		
**Sample models**	**Model size**	**No. of analysed parameters**	**No. of sampled sets**	**Mathematica Implementation (1 core)**	**Python Implementation (1 core)**	**Python Implementation (8 cores)**
The Goodwin system	3 ODEs	2	1000	40 (s)	70 (s)	20 (s)
		3	1000	40 (s)	20 (s)	6 (s)
		6	10,000	425 (s)	200 (s)	45 (s)
2-Layer PFB cascade	2 ODEs	4	1000	35 (s)	6 (s)	2 (s)
		4	20,000	700 (s)	245 (s)	30 (s)
		9	20,000	1000 (s)	365 (s)	44 (s)

A guide for running time (measured in seconds) for DYVIPAC using the Mathematica and Python-based implementations for different models specifications (PFB: postitive feedback).
